# Detrimental effects of branched-chain amino acids in glucose tolerance can be attributed to valine induced glucotoxicity in skeletal muscle

**DOI:** 10.1038/s41387-022-00200-8

**Published:** 2022-04-13

**Authors:** Christopher A. Bishop, Tina Machate, Thorsten Henning, Janin Henkel, Gerhard Püschel, Daniela Weber, Tilman Grune, Susanne Klaus, Karolin Weitkunat

**Affiliations:** 1 Department of Physiology of Energy Metabolism, German Institute of Human Nutrition Potsdam-Rehbruecke, Nuthetal, 14558 Germany; 2 University of Potsdam, Institute of Nutrition Science, Potsdam-Rehbruecke, Nuthetal, 14558 Germany; 3 Department of Molecular Toxicology, German Institute of Human Nutrition Potsdam-Rehbruecke, Nuthetal, 14558 Germany; 4 University of Potsdam, Institute of Nutrition Science, Nutritional Biochemistry Dept, Nuthetal, 14558 Germany; 5 University of Bayreuth, Faculty of Life Science, Department of Nutritional Biochemistry, Kulmbach, 95326 Germany

**Keywords:** Type 2 diabetes, Mouse, Homeostasis, Metabolic syndrome, Metabolic syndrome

## Abstract

**Objective:**

Current data regarding the roles of branched-chain amino acids (BCAA) in metabolic health are rather conflicting, as positive and negative effects have been attributed to their intake.

**Methods:**

To address this, individual effects of leucine and valine were elucidated in vivo (C57BL/6JRj mice) with a detailed phenotyping of these supplementations in high-fat (HF) diets and further characterization with in vitro approaches (C2C12 myocytes).

**Results:**

Here, we demonstrate that under HF conditions, leucine mediates beneficial effects on adiposity and insulin sensitivity, in part due to increasing energy expenditure—likely contributing partially to the beneficial effects of a higher milk protein intake. On the other hand, valine feeding leads to a worsening of HF-induced health impairments, specifically reducing glucose tolerance/insulin sensitivity. These negative effects are driven by an accumulation of the valine-derived metabolite 3-hydroxyisobutyrate (3-HIB). Higher plasma 3-HIB levels increase basal skeletal muscle glucose uptake which drives glucotoxicity and impairs myocyte insulin signaling.

**Conclusion:**

These data demonstrate the detrimental role of valine in an HF context and elucidate additional targetable pathways in the etiology of BCAA-induced obesity and insulin resistance.

## Graphical abstract

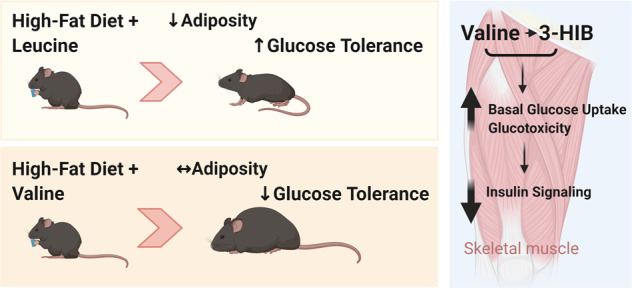

## Introduction

Conflicting data with regards to protein intake in metabolic health has been coined the protein paradox: positive anabolic effects of high protein intake are observed in intervention studies, but most epidemiological studies show an association of low protein diets with improved health [1]. Furthermore, the branched-chain amino acids (BCAA), leucine (Leu), valine (Val) and isoleucine (Ile), are essential amino acids whose role in metabolic health is rather conflicting—both detrimental and positive effects have been described depending on the context.

Increased circulating BCAA, deemed the “BCAA signature”, in obese and type-2-diabetic patients is a hallmark of disease [2–6], and impaired BCAA metabolism has been linked to a causal role in diabetes development [7]. This signature is also observed in genetic mouse models of obesity, where increased circulating levels of BCAA were shown to be due to impaired catabolism [8]. Further research into possible causal roles for BCAA in poor metabolic health outcomes demonstrated that a high BCAA mixture intake leads to obesity development and shortening of lifespan in mouse feeding studies [9]. High-fat diet (HF) feeding with BCAA supplementation appears to have a negative effect on insulin sensitivity [2], but protects from HF-induced weight gain due to aberrant lipolysis [10]. In line with that, restriction of BCAA in the diet—which implicated individual roles for Leu, Ile, and Val—[11–15] and pharmacological activation of the catabolic pathway result in improved metabolic health [8].

On the other hand, several studies demonstrated the efficacy of a high protein diet in promoting weight loss in both animals and humans [16, 17], and modulation of the carbohydrate-protein ratio to a higher protein content leads to improved health outcomes [18]. This seems to be replicated in an HF context, where a high protein supplementation has anti-obesogenic effects. Furthermore, HF related health impairments were alleviated by Leu or Ile supplementation, which protected from HF-induced weight gain and recovered glucose tolerance [19–21].

Taken together, these findings are rather contradictory, and although the specific contributions of Leu and Ile have been explored, little is known regarding the role of Val feeding in a high-fat context. Differential short-term effects of Leu versus Val supplementation have been observed [22] and reduction of Val from diet has modest metabolic effects [15]. However, clarifying long-term effects of individual BCAA supplementations is of importance for understanding the implications on whole-body metabolism. Recently, a Val-derived metabolite, 3-hydroxyisobutyrate (3-HIB) was shown to induce trans-endothelial fatty acid (FA) uptake and glucose intolerance [23] and was associated with hyperglycemia/type-2-diabetes [24]. Therefore, we sought to characterize the individual roles of the BCAA, Val or Leu, in a long-term HF diet feeding study, in comparison to the effects of a high protein supplementation via casein (milk protein). Herein, we highlight the negative role of Val in glucose homeostasis with further examination into the pathways involved in the etiology of Val induced glucose intolerance.

## Research design and methods

### Animals and experimental setup

C57BL/6JRj male mice were purchased from Janvier Labs, maintained on 12-h light/dark cycle, and group-housed. At 12 weeks of age mice were randomly assigned into groups of equivalent body weight and experimental diets were given for 4 or 20 weeks with *ad libitum* access to food and water. Control low- and high-fat diets were used along with experimental high-fat diets supplemented with higher casein, Leu or Val (Table 1). Two hour fasted animals were killed by cervical dislocation; following blood collection, tissues were isolated, weighed, and snap frozen in liquid nitrogen. All experiments were approved by the ethics committee of the Ministry for Environment, Health, and Consumer Protection of Brandenburg, Germany (approval no. 2347-17-2018). Following 4 weeks of intervention, 2 h fasted mice received i.p. injection of insulin (0.75 U/kg body weight). After 30 mins, mice were killed by cervical dislocation and tissues snap frozen.

**Table 1:** Composition of semisynthetic experimental low-fat (LF) and 40 Energy% high-fat (HF) diets supplemented with milk protein (HFMP), leucine (HFL), or valine (HFV) produced by ssniff Spezialdiäten GmbH.

Components	LF	HF	HFMP	HFL	HFV
	(g/kg)	(g/kg)	(g/kg)	(g/kg)	(g/kg)
Casein^*^	140	140	280	140	140
**Wheat starch**	466.5	299.5	159.5	249.5	249.5
**Maltodextrin**	100	100	100	100	100
**Dextrose**	50	50	50	50	50
**Sucrose**	100	100	100	100	100
**Lipids**	43	210	210	210	210
**Cellulose**	50	50	50	50	50
**L-Leucine**	–	–	–	50	–
**L-Valine**	–	–	–	–	50
**Mineral mixture**	35	35	35	35	35
**Vitamin mixture**	10	10	10	10	10
**Choline bitartrate**	2.5	2.5	2.5	2.5	2.5
**L-Cysteine**	3	3	3	3	3
**Energy content (kJ/g)**	16.5	20.1	20.1	20.1	20.1

^*^Calcium Caseinate 380 was provided by NZMP.

#### Metabolic phenotyping

Body weight and composition were taken weekly. Fat mass was measured using nuclear magnetic resonance spectrometer EchoMRI^TM^-Analyzer (Echo Medical Systems) and lean mass calculated as difference between fat mass and body weight. Indirect calorimetry measurements were measured in week 12 using the PhenoMaster System (TSE Systems GmbH). Energy intake was calculated by multiplying food intake with the energy content of diets determined from bomb calorimetry, as previously described [25].

#### Tolerance tests

Oral glucose tolerance tests (oGTT) were performed in week 6 and 16 of intervention after 16 h fast with a 20% glucose solution (2 g/kg body weight). Blood glucose and plasma insulin were measured using a glucometer (Bayer) and mouse ultrasensitive insulin ELISA (ALPCO-80-INSMSU-E01), respectively. i.p. insulin tolerance test (ITT) was performed after 2 h fasting at week 16 (0.75 U/kg body weight).

### Analytic procedures

#### Plasma and tissue analysis

Liver and quadriceps TAG were measured with TAG determination kit (Sigma Aldrich). Briefly, tissues were lysed (10 mM sodium phosphate buffer (pH 7.4),1 mM EDTA, 1% polyoxyethylene [10] tridecyl ether) and subsequently measured. Glycogen content was measured using Starch Analysis kit (R-BioPharm AG) after extraction with 0.1 N NaOH. Quadricep DAGs were measured according to manufacturer’s protocol (ab242293, Abcam). Relative lipid content was quantified in randomly chosen microphotographs of H&E-stained liver sections containing no blood vessels (central veins or portal fields) using ImageJ software as described [26]. Lipid droplet areas were defined as sum of histogram data in the range of 152-255 and calculated relative to the overall density per field.

Circulating BCAA concentrations were determined with LC-MS/MS as described with some modifications [27]. Briefly, 10 µL plasma samples were mixed with 40 µL of an internal standard solution (62.5 µM of isoleucine-^13^C_6_,^15^N, leucine-^13^C_6_,^15^N, and valine-^13^C_5_ in 90% acetonitrile). Protein was precipitated by storing samples at −20 °C for 10 min and subsequent centrifugation for 10 min at 17,000 g, 4 °C. 2 µl of sample supernatant was then injected into LC-MS/MS system.

Plasma levels of 3-HIB and ketone bodies (α-hydroxybutyrate, β-hydroxybutyrate, and acetoacetate) were measured by GC-MS/MS after methylchloroformate derivatization at Bevital AS (Bergen, Norway; http://www.bevital.no) as described [28]. Lactate was measured using the Cobas Mira chemistry analyzer (Roche) with the appropriate reagent kits.

#### Quantitative real-time PCR (qRT-PCR)

mRNA analysis was performed as described [29]. RNA was extracted (TriFast reagent), followed by DNase treatment and cDNA synthesis according to suppliers’ protocol (LunaScript RT SuperMix, NEB). Primer sequences supplied in Supplementary Table 1. Gene expression was calculated as ddCT, using normalizer genes as specified and expressed relative to the respective control group, as value of 1.

#### Western blotting

Total protein was isolated using RIPA buffer containing Halt Protease/Phosphatase Inhibitor Cocktail (ThermoFisher) as previously described [29]. See Supplementary Table 2 for antibodies used. Membrane fractionation was performed as described [30]. Densitometry was determined using ImageJ and normalized to respective control group.

### Cell Culture—C2C12 Myotube Cultures

C2C12 cells were cultured in DMEM with 10% FBS and 1% penicillin–streptomycin as described [31]. At 90% confluency, differentiation of cells was induced for 7 days with DMEM containing 3% horse serum. Cells were treated with either L-Valine (Sigma, V-0513) or Sodium-β-hydroxyisobutyrate (3-HIB) (Sigma, 36105) in presence of complexed BSA: palmitic acid (125 µM BSA: 250 µM PA) with BSA control for the specified duration and concentration in the serum starvation media. For insulin stimulation, media was changed to insulin free, 2-h prior to stimulation (15 min) with 100 nM insulin. Glucose Uptake-Glo Assay (Promega, J1341) was used to determine basal and insulin stimulated glucose uptake after 48 h. GLUT1 inhibitor studies were performed at indicated time points with BAY-876 (R&D, 6199).

### Statistical analysis

Statistical calculations were performed using GraphPad Prism 8. All data are represented as mean ± SEM. Animal sample number size was determined based on previous experiments [19]. For certain experimental procedures (i.e., qRT-PCR, plasma & tissue analysis) investigators, including technicians, were blinded to group allocation. Comparisons for normality were determined between groups before further analysis. Normally distributed data were analyzed using an ordinary one-way ANOVA with Bonferroni’s post hoc and Kruskal–Wallis tests for non-normally distributed. Grubbs outlier tests were performed to determine outliers. Pearson correlation coefficient *R*^2^ is used to describe correlations. Differences with *p* < 0.05 were considered statistically significant.

## Results

### Valine feeding leads to a worsening of fat accumulation under HF conditions

**Fig. 1:**
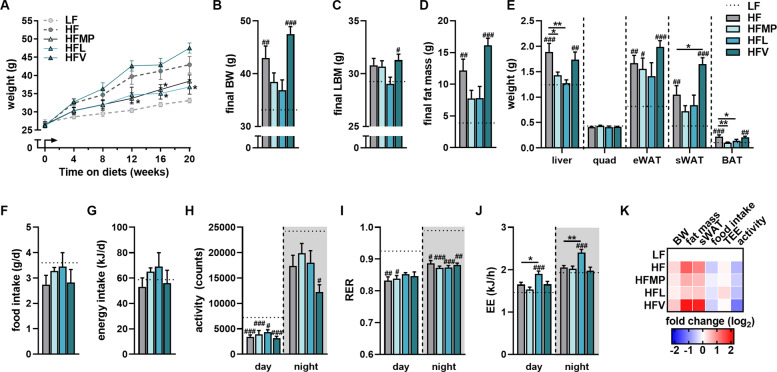
Valine feeding drives adiposity under high-fat diet conditions. **A** Body weight development in male C57BL/6 JRj mice fed low-fat (LF), high-fat (HF), or experimental HF diets supplemented with milk protein (HFMP), leucine (HFL), or valine (HFV) for 20 weeks (*n* = 12). **B** Final body weight at 20 weeks (*n* = 12). **C** Final calculated lean body mass (*n* = 12). **D** Final fat mass determined with NMR (*n* = 12). **E** Final tissue weights for liver, muscle and adipose tissue depots (*n* = 12). **F** Average food intake per day over 24 h (*n* = 5–7). **G** Calculated energy intake per day from average food intake and energy content of diets. (*n* = 5–7). **H** Average locomotor activity (beam breaks) x1000 per 12 h period (day and night phase) (*n* = 7). **I** Respiratory exchange ratio (RER; *n* = 8). **J** Average energy expenditure (EE) per hour per 12 h period (day and night phase) (*n* = 8). **K** Heatmap of phenotypic effects. Data are mean + SEM, LF is represented as dotted line. *p < 0.05; **p < 0.01; ***p < 0.001 compared to HF and ^#^*p* < 0.05; ^##^*p* < 0.01; ^###^*p* < 0.001 compared to LF. BW body weight, LBM lean body mass, eWAT epididymal white adipose tissue, sWAT subcutaneous white adipose tissue, BAT brown adipose tissue, quad quadriceps muscle.

To further clarify differential roles of BCAA in metabolic health we performed a 20-week HF feeding study with individual BCAAs (Leu, HFL; Val, HFV) in comparison with a milk protein rich diet (HFMP) which is a high source of the BCAA (Table 1). Feeding of HFMP and HFL resulted in a lower body weight gain compared to HF (Fig. 1A, B) due to a reduced fat mass accumulation (Fig. 1D). On the contrary, HFV did not prevent the HF-induced body weight gain and rather led to an amassing of fat, most significantly subcutaneous white adipose tissue (sWAT) (Fig. 1A–E). These results were evident already after 4 weeks of intervention, where the HFV group had the highest final fat mass, sWAT and epidydimal WAT (eWAT) weights (Fig. S1A–E). The differences in body weight gain are likely not due to food or energy intake, as these showed no significant differences between the groups (Fig. 1F, G). Further characterization with indirect calorimetry showed no differences in locomotor activity (Fig. 1H) or in respiratory exchange ratio (RER) between high-fat fed groups (Fig. 1I). Additionally, energy expenditure (EE) was significantly higher in HFL mice during day and night compared to HF (Fig. 1J). While supplementation of Leu or milk protein provided a certain protection from deleterious effects of HF diet with regards to fat accumulation, these were not apparent after HFV feeding (Fig. 1K).

### Glucose intolerance is induced after long-term HF-valine feeding

**Fig. 2:**
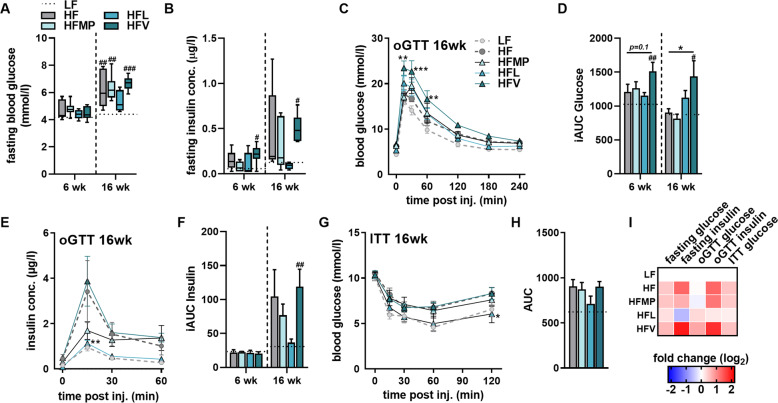
Glucose clearance is impaired after long-term valine feeding, independent of glucose-stimulated insulin secretion. **A** Sixteen-hour fasting blood glucose in male C57BL/6 JRj mice fed low-fat (LF), high-fat (HF) or experimental HF diets supplemented with milk protein (HFMP), leucine (HFL) or valine (HFV) for 6 or 16 weeks (*n* = 6–8). **B** Sixteen-hour fasting insulin at 6 or 16 weeks (*n* = 6–8). **C** Glucose measurements during an oral glucose tolerance test (oGTT; oral gavage 2 g/kg body weight) after 16 h fast at 16 weeks of feeding (*n* = 6–8). **D** Incremental area under the curve (iAUC) of glucose levels during oGTT at week 6 or 16 of experiment (*n* = 6–8). **E** Insulin measurements during oGTT after 16 weeks of feeding (*n* = 6). **F** Incremental area under the curve (iAUC) of insulin levels during oGTT at week 6 or 16 of experiment (*n* = 6–8). **G** Glucose measurements during an insulin tolerance test (ITT; i.p. 0.75U insulin) after 2 h fast at 16 weeks (*n* = 6). **H** Area under the curve (AUC) of glucose levels during i.p. ITT (*n* = 6). **I** Heatmap of phenotypic effects. Plasma data (**A**, **B**) are expressed as interleaved box and whiskers (min to max) plots. All other data are shown as mean + SEM. LF is represented as dotted line. *p < 0.05; **p < 0.01; ***p < 0.001 compared to HF and ^#^*p* < 0.05; ^##^*p* < 0.01; ^###^*p* < 0.001 compared to LF.

In contrast to Leu, little is known about the specific effects of valine on insulin sensitivity and glucose tolerance. Fasting blood glucose and insulin levels tended to be lower in the HFL group after 16 weeks, which was not observed in HFMP and HFV-fed mice (Fig. 2A, B). The calculated HOMA-IR reflects the fasting insulin levels, with no specific differences between HF and HFMP or HFV (data not shown). As apparent in the oGTT at 6 weeks, and exacerbated after 16 weeks, HFV showed a strong delay in glucose clearance, as seen in the incremental AUC (iAUC) (Fig. 2C, D), with no difference between HF and HFV feeding in insulin secretion, suggesting peripheral insulin resistance (IR) in HFV mice (Fig. 2E, F). HFL feeding had no significant effects on blood glucose clearance compared to HF (Fig. 2C, D) but displayed insulin values similar to LF fed mice (Fig. 2E, F), which is supported by an insulin response similar to that of LF fed mice in the i.p. ITT (Fig. 2G, H). There were no significant differences between HF and HFMP throughout these studies. These data again support the suggested protective effects of Leu, and highlight a causal role for Val in glucose intolerance (Fig. 2I).

### HF-valine feeding shunts BCAA catabolism towards skeletal muscle and increases circulating 3-hydroxyisobutyrate (3-HIB) levels

**Fig. 3:**
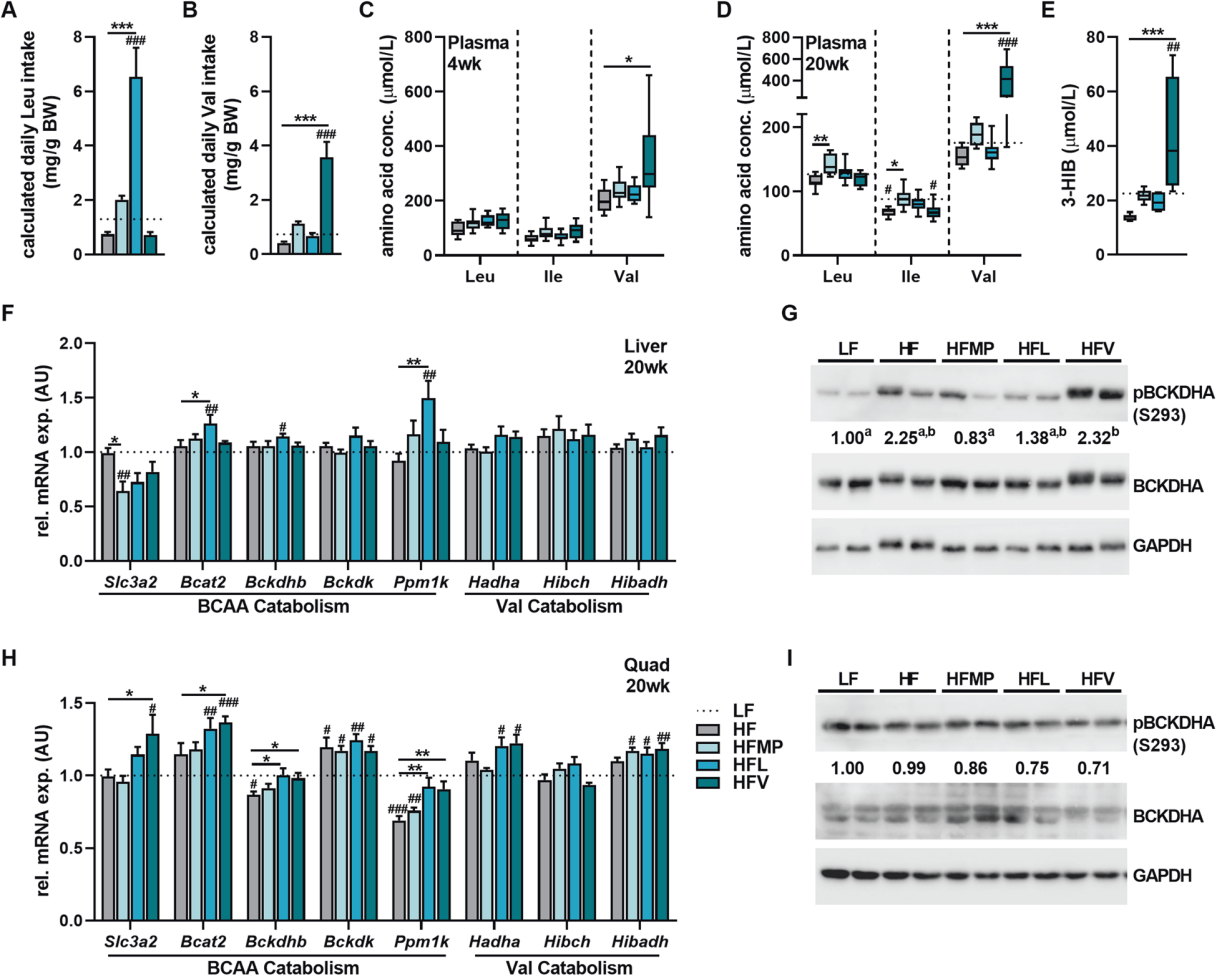
Accumulation of valine and its metabolite 3-hydroxyisobutyrate after valine supplementation. **A** Calculated daily Leu intake for male C57BL/6 JRj mice fed low-fat (LF), high-fat (HF), or experimental HF diets supplemented with milk protein (HFMP), leucine (HFL), or valine (HFV) for 20 weeks (*n* = 8). **B** Calculated daily Val intake (*n* = 8). **C** Two-hour fasting plasma BCAA levels after 4 weeks (*n* = 8). **D** 2 h fasting plasma BCAA levels after 20 weeks (*n* = 8). **E** Two-hour fasting plasma 3-hydroxyisobutyrate (3-HIB) levels at 20 weeks (*n* = 6). **F** qPCR of key genes in BCAA oxidation normalized to LF in liver at 20 weeks with *B2m* as reference gene (*n* = 8). **G** Representative western blot of BCKDHA phosphorylation in liver normalized to LF (*n* = 6). **H** qPCR of key genes in BCAA oxidation normalized to LF in quadriceps (quad) muscle at 20 weeks with *B2m* as reference gene (*n* = 8). **I** Representative western blot of BCKDHA phosphorylation in quad normalized to LF (*n* = 6). Plasma data (**C**–**E**) are expressed as interleaved box and whiskers (min to max) plots. All other data are shown as mean + SEM except for western blot quantification where only mean is indicated below representative blots. LF is represented as dotted line. *p < 0.05; **p < 0.01; ***p < 0.001 compared to HF and ^#^*p* < 0.05; ^##^*p* < 0.01;^###^*p* < 0.001 compared to LF. Different letters represent significant difference between groups in Western blots. BCAA branched-chain amino acids, FA fatty acids.

We next aimed to characterize the molecular effects of increased Val intake regarding BCAA catabolism. The calculated daily Leu and Val intake (using diet composition and food intake data) showed an ~6-fold increased Leu and 4-fold increased Val intake by HFL and HFV feeding, respectively, compared to the HF group. Of note, HF feeding led to a decreased Leu and Val intake compared to the LF control group, while HFMP increased both Leu and Val intake by about 50% compared to HF (Fig. 3A, B). HFMP feeding led to marginally higher plasma concentrations of Leu and Ile, compared to HF, which was evident after 20 but not yet after 4 weeks, while HFL feeding induced only a mild increase (trending, *p* = *0.12)* in circulating Leu. In addition, there was a tendency for increased ketone bodies (acetoacetate—AcAc; beta-hydroxybutyrate—β-HB) which are the end products of leucine catabolism (Fig. S2G). Strikingly, HFV mice displayed a 4-fold increase in circulating Val, with unaffected levels of Leu and Ile (Fig. 3D) already after 4 weeks (Fig. 3C). Circulating Val concentrations at 20 weeks also correlated with final fat mass (*R*^2^ = 0.371; *p* < 0.02), sWAT weight (*R*^2^ = 0.417; *p* < 0.008) at 20 weeks, and iAUC of the blood glucose from the oGTT (*R*^2^ = 0.535; *p* < 0.05) at 16 weeks. Additionally, HFV feeding led to increased circulating levels of the Val-derived metabolite, 3-HIB (Fig. 3E). Liver expression of BCAA catabolic genes were mostly unaffected by HFMP and HFV. However, HFL increased gene expression of branched-chain amino transaminase (Bcat2) and protein phosphatase 1 K (Ppm1k), which activates branched-chain keto-acid dehydrogenase (BCKDHA) via dephosphorylation (Fig. 3F). Both HFL and HFMP led to reduced phosphorylation levels of BCKDHA, while HF and HFV showed increased inhibitory levels of phosphorylation (Fig. 3G). These increases in inhibitory phosphorylation of BCKDHA in HF and HFV are likely due to the fact that both groups were not protected from HF-induced hepatic lipid accumulation reflecting an increased gene expression of the fatty acid transporter Cd36. In contrast, both liver fat, glycogen, and liver *Cd36* expression was not different from the LF-control in the HFMP and HFL fed animals (Fig. S2A–C). After HFV feeding, BCAA catabolism appeared to be shunted towards skeletal muscle, as these mice showed increased expression of transport (*Slc3a2*) as well as BCAA catabolic genes (*Bcat2, Bckdhb, Ppm1k,* and *Hadha*) with no significant differences of the inhibitory phosphorylation of BCKDHA (Fig. 2H, I).

### HF-valine feeding compromises skeletal muscle insulin signaling

**Fig. 4:**
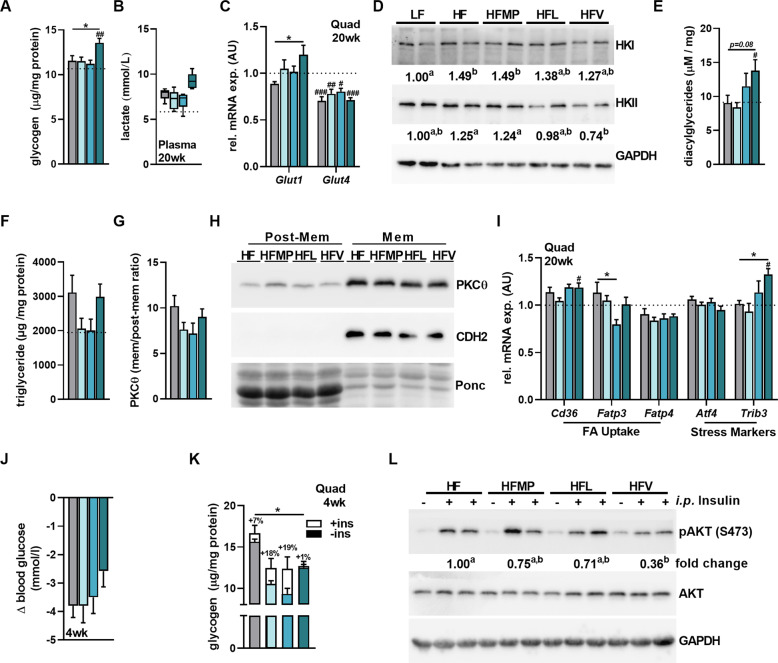
Valine feeding leads to impaired skeletal muscle insulin signaling. **A** Quadriceps (quad) glycogen levels in male C57BL/6 JRj mice fed low-fat (LF), high-fat (HF), or experimental HF diets supplemented with milk protein (HFMP), leucine (HFL), or valine (HFV) for 20 weeks (*n* = 8). **B** Two-hour fasting plasma lactate levels at 20 weeks (*n* = 6). **C** qPCR of key genes in glucose transport normalized to LF in quad at 20 weeks with *Hprt* as reference gene (*n* = 8). **D** Representative western blot of Hexokinase I & II in quad at 20 weeks normalized to LF (*n* = 6). **E** Quad diacylglyceride levels at 20 weeks (*n* = 8). **F** Quad triglyceride levels at 20 weeks (*n* = 8). **G** Ratio of PKCθ localization from membrane (mem) to post membrane (post-mem) fraction (*n* = 6). **H** Representative western blot of PKCθ membrane fractionation normalized to LF. **I** qPCR of key genes in FA uptake and ER stress normalized to LF with *Hprt* as reference gene (*n* = 8). **J** Delta blood glucose after 30 min insulin stimulation from 4 week fed mice (*n* = 8). **K** Quad glycogen levels after 30 min insulin stimulation from 4 week fed mice (*n* = 8). **L** Representative western blot of insulin stimulated (30 min) AKT phosphorylation in quad of mice fed for 4 weeks (*n* = 8). Fold change in Western blots (**L**) refers to insulin induced change normalized to HF. Plasma data (**B**) are expressed as interleaved box and whiskers (min to max) plots. All other data are shown as mean + SEM except for western blot quantification where only mean of signal or fold change is indicated below representative blots. LF is represented as dotted line. *p < 0.05; **p < 0.01; ***p < 0.001 compared to HF and ^#^*p* < 0.05; ^##^*p* < 0.01; ^###^*p* < 0.001 compared to LF. Different letters represent significant difference between groups in Western blots. FA, fatty acids.

As skeletal muscle accounts for roughly 70% of insulin stimulated glucose clearance, we sought to address the cause for Val-induced glucose intolerance in muscle. At 20 weeks, basal muscle glycogen and circulating lactate were highest in HFV-fed mice (Fig. 4A, B). Gene expression of the non-insulin dependent glucose transporter, *Glut1*, was higher in HFV-fed mice compared to HF, while *Glut4* expression was similarly downregulated in all HF groups compared to LF (Fig. 4C). With no effects on hexokinase (HK) I protein expression, HKII protein expression was downregulated after HFV feeding (Fig. 4D). Further indicative of increased intracellular glucose-6-phosphate (G6P) levels, diacylglycerides (DAG) were highest in the HFV group (Fig. 4E), with no differences in TAG levels (Fig. 4F). Membrane localization of PKCθ, a regulator of IRS1 which is typically activated by DAGs [32], showed no differences (Fig. 4G, H) and was more reflective of the TAG accumulation. Moreover, we observed no differences in IRS1 phosphorylation (Fig. S3A) or other upstream mediators such as JNK and mTOR (Fig. S3B, C). HFV feeding did, however, lead to an upregulation of gene expression for the glucotoxicity stress marker Tribbles 3 (*Trib3*), which has direct inhibitory effects on AKT [33] (Fig. 4I). We then explored whether acute insulin signaling in the muscle was altered after 4 weeks of supplementation. HFV-fed mice tended to a lower blood glucose clearance, and exhibited significantly lower glycogen formation (Fig. 4J, K) and decreased phosphorylation of AKT in muscle after acute insulin stimulation (Fig. 4L).

### 3-HIB-induced skeletal muscle glucotoxicity impairs insulin signaling in vitro

**Fig. 5:**
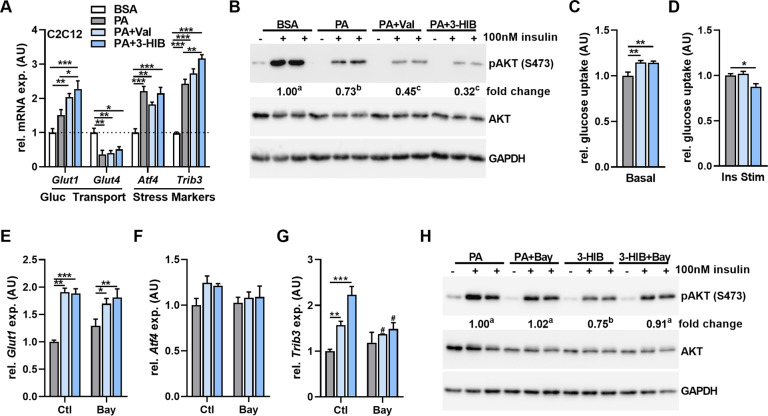
Valine-metabolite, 3-hydroxyisobutyrate drives basal glucose uptake and glucotoxicity-mediated impairment of myotube insulin signaling. **A** qPCR of glucose transporters and stress markers in C2C12 mouse myotubes after treatment with 2 mM Val or 3-HIB in presence of 250 µM PA for 24-h normalized to BSA control with *B2m* as reference gene (*n* = 4). **B** Representative western blot of insulin stimulated (15 min) AKT phosphorylation in differentiated C2C12 mouse myotubes after treatment with 2 mM valine (Val) or 3-HIB for 48-h normalized to BSA control (*n* = 4). **C** Relative basal glucose uptake in differentiated C2C12 mouse myotubes after treatment with 2 mM valine (Val) or 3-HIB for 48-h normalized to PA (*n* = 8). **D** Relative insulin stimulated (30 min) glucose uptake in differentiated C2C12 mouse myotubes after treatment with 2 mM valine (Val) or 3-HIB for 48-h normalized to PA (*n* = 8). **E**–**G** qPCR of glucose transporter and stress markers in C2C12 mouse myotubes after treatment with 2 mM Val or 3-HIB in presence of 250 µM PA and 25 nM BAY-876 (Bay) for 24 h normalized to PA-Ctl with *B2m* as reference gene (*n* = 4). **H** Representative western blot of insulin-stimulated (15 min) AKT phosphorylation in differentiated C2C12 mouse myotubes after treatment with 2 mM valine (Val) or 3-HIB in presence of 25 nM BAY-876 normalized to PA control (*n* = 4). Fold change in Western blots (**B**, **H**) refers to insulin-induced change normalized to BSA control (**B**) or PA control (**H**). Data are shown as mean + SEM except for western blot quantification where only mean of fold change is indicated below representative blots. *p < 0.05; **p < 0.01; ***p < 0.001 compared to respective control and. ^#^*p* < 0.05 comparing Ctl to Bay treated groups. Different letters represent significant difference between groups in Western blots. BSA bovine serum albumin, PA palmitic acid, Bay BAY-876.

To test for a direct effect of Val / 3-HIB in driving the apparent impaired muscle insulin signaling, C2C12 mouse myotubes were used. Treatment with both Val or 3-HIB (2 mM) led to significant increases in *Glut1* and *Trib3 gene* expression after 24 h under lipid-loaded conditions (presence of 250 µM palmitic acid—PA) (Fig. 5A). Both treatments also led to significant reductions in AKT phosphorylation after insulin stimulation (Fig. 5B). Additionally, it was possible to confirm that treatment with Val and 3-HIB led to a significant upregulation of basal glucose uptake compared to PA treatment alone; while only 3-HIB treatment led to a reduction in insulin-stimulated glucose uptake (Fig. 5C, D). GLUT1 inhibitor (Bay-876) treatment, while having no impact on *Glut1* gene expression, resulted in a reversal of the *Trib3* upregulation after Val or 3-HIB treatment (Fig. 5E–G). Finally, insulin stimulation of differentiated C2C12 treated for 24 h with 3-HIB and GLUT1 inhibitor resulted in a rescue of the 3-HIB-induced decrease of AKT signaling (Fig. 5H).

## Discussion

Increased protein intake in relation to obesity and metabolic health, to date, remains controversial, because beneficial and detrimental health effects of high protein diets have been both been described. To this point, studies have highlighted the involvement of the essential BCAA in the contended role of protein intake on metabolic health. Dietary protein contains around 20% BCAA [34], and we have previously shown differential acute effects of Leu and Val on hepatic FA metabolism [22]. Here we examined the long-term physiological effects of either Leu or Val supplementation on the development of diet-induced obesity and associated metabolic disturbances, in comparison to the supplementation of milk protein (casein), a natural source of protein containing ~20% BCAA. Using whole-body metabolic phenotyping and molecular characterization, we show that, while milk protein and Leu have protective metabolic effects, Val induces a disturbance in glucose tolerance, which is driven in part by persistently elevated circulating 3-HIB levels mediating a skeletal muscle IR by driving basal glucose uptake resulting in glucotoxicity inhibition of AKT signaling, findings confirmed with in vitro studies.

It has been previously posited that the beneficial aspects of a higher protein intake on body weight development is at least in part mediated by a higher Leu intake—although supplementation of other amino acids also appear to protect from some HF-induced effects. Importantly, we confirm that Leu and milk-protein prevented HF-related adiposity, in line with what has been previously shown in mouse studies [16, 19, 21, 35]. The protective effects of Leu on body weight and fat mass could be attributed to an increased EE—which has been suggested to be due to an increase in browning/heat production [36]. Further, both HFL and HFMP feeding protected mice from ectopic hepatic lipid accumulation, in part by decreasing hepatic FA transport, confirming previous results [19]. Others highlighted the beneficial role in adipose tissue browning, as another possible contributing factor in the protection against diet-induced obesity, which was apparent after supplementation with Leu and Ile individually [21]. Although both HFL and HFMP fed groups were protected from increased adiposity, only HFL feeding had beneficial effects on insulin sensitivity and hyperglycemia/insulinemia. Several studies have already demonstrated the effects of Leu supplementation on glucose homeostasis, showing either no change or improved responses in glucose or insulin tolerance tests [20]. It is reported that leucine treatment acutely induces insulin secretion; however, as discussed by Zhang et al., this effect is possibly secondary to the improvements in insulin sensitivity / glycemic control due to a long-term adaptive response [37]. Notably, however, results from previous human and mouse studies provide robust evidence suggesting that HF-BCAA supplementation, as a complex mixture, results in IR but protects from HF-induced obesity due to an elevated lipolysis [2, 10], while others showed no difference on IR or adiposity in both an HF diet as well as an HF-high sucrose diet [38]. As these are inconsistent with the findings on Leu and Ile supplementation, this might indicate that there is a compensatory and critical effect via Val in driving IR.

Only recently, the role of Val in metabolic health has been addressed, albeit indirectly. Protein restriction as a means to improve metabolic health was suggested to be mediated via a reduction of BCAA [39]. This was further clarified by showing that long-term restriction of Val or Ile individually under HF conditions restored metabolic health to DIO mice [15]. Here, we demonstrate with in-depth phenotyping that HF feeding with Val supplementation had no effect on body weight development, food intake, or EE; however, HFV fed mice presented with IR, an apparent impaired glucose clearance with no differential effects on insulin secretion during an oGTT—further highlighting a detrimental role for Val. A possible explanation for the current conflicting literature of BCAA in obesity/IR development could be due to the apparent differential effects of Leu and Val on metabolic health under HF conditions. This demonstrates the importance of dietary protein quality, as Val and Leu appear to have counteracting effects in controlling glucose and insulin homeostasis. To an extent, these seemingly counteracting roles of Val and Leu in glucose homeostasis could explain why milk protein feeding only exerted beneficial effects on adiposity and not insulin sensitivity; however, further research is required to complement this hypothesis.

Pathological increases in circulating BCAA have been recapitulated in genetic animal models of obesity and IR (i.e., ob/ob mice and Zucker fatty rats), which have been attributed to a reduction of BCAA catabolic genes in certain tissues, as well as decreased activity of liver BCKDH [40]. Approximately 67% of whole-body BCAA oxidation occurs in muscle and liver [41]. The first step of BCAA oxidation is catalyzed by BCAT, followed by the irreversible action of BCKDH, a flux-controlling step regulated by opposing negative phosphorylation by BCKDH kinase (BCKDK) and PPM1K [42]. While in our study, HF alone did not lead to increased fasting circulating BCAA levels, which is consistent with DIO mouse models [2], HFV led to a reduction in hepatic BCAA catabolism, and catabolism appears rather shunted towards skeletal muscle—something that is evident in HF-BCAA fed mice [38]. The impaired BCAA catabolism in the liver is likely a result of HF-induced lipotoxicity, as HFV feeding does not protect from ectopic lipid accumulation in the liver. This shift in catabolism may play a role in the accumulation of Val and its metabolite 3-HIB in the plasma of the HFV-fed mice.

Several models for BCAA-induced IR have been put forth, including 3-HIB driven lipotoxicity (i.e., DAG accumulation) and PKCθ-mediated IR [23]. Inhibitory phosphorylation of IRS1 by PKCs, JNK, or mTOR have all been suggested as mediators in IR [43]; however, we observed no effects with that regard in skeletal muscle. Nevertheless, AKT phosphorylation was decreased after insulin stimulation in HFV mice, suggesting another mechanism. While we show an accumulation of DAG in muscle, another possible driving factor for BCAA-related IR is the apparently increased basal glucose uptake in connection with Val supplementation. At 20 weeks of feeding, HFV appeared to promote accumulation of glycogen levels in the muscle and increased circulating lactate, likely due to intracellular hyperglycemia. HFV feeding resulted in a strong induction of *Glut1* expression, the basal glucose transporter. Overexpression of this transporter has been associated with the development of IR via increased intracellular glucose [44].

Hyperglycemia has a number of negative implications on metabolism, including DAG accumulation [45]. We suggest that a higher basal glucose flux and 3-HIB induced fatty acid uptake could be driving the buildup of DAG in the skeletal muscle of Val-fed mice; however, this did not lead to increased PKC translocation or IRS1 phosphorylation. Other inhibitors further downstream in the insulin signaling cascade are likely mediating this IR, for example, AKT inhibition has been previously reported via PH domain and leucine-rich repeat protein phosphatase (PHLPP), protein phosphatase 2 (PP2A) or TRIB3 [46, 47]. Obesity and type-2-diabetes in humans, as well as high-fat feeding in mice, have been shown to exhibit increased expression of Trib3 in skeletal muscle [48, 49], while Trib3 knockout mice were protected from glucose-induced IR, suggesting TRIB3 to be a critical response factor in nutrient excess [33, 47, 50]. We clearly show that Val supplementation led to an induction of Trib3 expression, which is further supported using an in vitro model of differentiated mouse myotubes, where Val and its metabolite 3-HIB actually lead to an induction of *Glut1* expression and basal glucose uptake. Both treatments induced a reduction in AKT phosphorylation after an acute insulin stimulation, as well as *Trib3* expression—apparently dependent on Glut1, as these effects were mitigated after inhibition of Glut1 activity with the GLUT1-selective inhibitor BAY-876. Taken together, we posit that the Val mediated intracellular hyperglycemia due to increased basal glucose transport may lead to glucotoxicity, activation of related pathways (i.e., DAG accumulation, AGE formation, and the hexosamine biosynthesis [45]), and subsequent inhibition of insulin signaling by action of TRIB3.

## Conclusions

Using individual BCAA supplementations we could confirm that long-term HFL feeding leads to protective effects with regards to diet induced adiposity and insulin sensitivity which is similar to the effects of high protein diet. In contrast, Val supplementation did not improve but rather worsened HF-induced health impairments, specifically reducing glucose tolerance/insulin sensitivity. This was linked led to an accumulation of circulating Val and its metabolite 3-HIB, which are driving the hyperglycemia-induced skeletal muscle IR. By mainly focusing on the individual effects of the BCAA, Leu, and Val, we were able to reveal substantial differences in the metabolic effects of the two and show that the combination of high Val and high-fat intake plays a harmful part in the development and worsening of metabolic disorders.

## Supplementary information

Supplemental Materials

Supplementary Figure 1

Supplementary Figure 2

Supplementary Figure 3

Supplementary Table 1

Supplementary Table 2

Supplementary Table 3

## Data Availability

All primary data within this study are available upon reasonable request.
