# Supplementary material for: Detrimental effects of branched-chain amino acids in glucose tolerance can be attributed to valine induced glucotoxicity in skeletal muscle
Source: Nutr Diabetes. 2022 Apr 13;12:20. doi: 10.1038/s41387-022-00200-8 (PMC9008040; doi:10.1038/s41387-022-00200-8)
Supplement: Supplementary file 1 — Supplemental Materials [file 41387_2022_200_MOESM1_ESM.docx]

**Supplemental Materials**

**Supplementary Table 1.** Primers used for qPCR at final concentration of 2.5µM

**Supplementary Table 2.** Antibodies used for western blots at dilution of 1:1000 in 5% BSA.

**Supplementary Table 3.** 2hr fasting plasma amino acid levels (µM) in male C57BL/6J fed high-fat (HF) or experimental HF diets supplemented with milk protein (HFMP), leucine (HFL) or valine (HFV) for 20 weeks (n = 8).

**Supplementary Figure Legends**

**Figure S1. 4 week supplementation of Valine sufficient for adipose tissue expansion.**

(A) Body weight development in male C57BL/6J mice fed low-fat (LF), high-fat (HF) or experimental HF diets supplemented with milk protein (HFMP), leucine (HFL) or valine (HFV) for 4 weeks (n = 9-10).

(B) Final body weight at 4 weeks (n = 9-10).

(C) Final calculated lean body mass (n = 9-10).

(D) Final fat mass determined with NMR (n = 9-10).

(E) Final tissue weights for liver, muscle and adipose tissue depots (n = 9-10). Data are mean + SEM, LF is represented as dotted line. *p < 0.05; **p < 0.01; ***p < 0.001 within groups. BW, body weight; LBM, lean body mass; eWAT, epidydimal white adipose tissue; sWAT, subcutaneous white adipose tissue; BAT, brown adipose tissue; quad, quadriceps muscle.

**Figure S2. Effects of BCAA supplementation on key hepatic metabolic pathways**

(A) Relative liver fat content as determined by H&E staining in male C57BL/6J fed high-fat (HF) or experimental HF diets supplemented with milk protein (HFMP), leucine (HFL) or valine (HFV) for 4 weeks (n = 8).

(B) Liver triglycerides (n = 8).

(C) Liver glycogen (n = 8).

(D) qPCR of key genes in glucose metabolism normalized to LF in livers at 20 weeks with *B2m* as reference gene (n = 8).

(E) qPCR of key genes in FA uptake / β-oxidation normalized to LF in livers at 20 weeks with *B2m* as reference gene (n = 8).

(F) Representative western blot of FA transport protein CD36 in liver at 20 weeks normalized to LF (n = 6).

(G) 2hr fasting plasma ketone bodies levels at 20 weeks (n = 6).

(H) qPCR of key genes in inflammation / fibrosis normalized to LF in livers at 20 weeks with *B2m* as reference gene (n = 8).

(I) Representative western blot of insulin stimulated (30min) AKT phosphorylation in liver of mice fed for 4 weeks. (n = 6)

Fold change in Western blots (I) refers to insulin induced change normalized to HF.

Data are mean + SEM, LF is represented as dotted line. *p < 0.05; **p < 0.01; ***p < 0.001 within groups and #p < 0.05; ##p < 0.01; ###p < 0.001 compared to LF. Different letters represent significant difference between groups in western blots. FA, fatty acids, GNG, gluconeogenesis, α-HB, α-hydroxybutyrate; β-HB, β-hydroxybutyrate; AcAc, acetoacetate

**Figure S3. Key signaling in quadriceps is unaffected by BCAA supplementation.**

(A) Representative western blot of IRS1 phosphorylation in quadriceps muscle (quad) of male C57BL/6J mice fed low-fat (LF), high-fat (HF) or experimental HF diets supplemented with milk protein (HFMP), leucine (HFL) or valine (HFV) for 20weeks (n = 6).

(B, C) Western blotting analysis of JNK and mTOR signaling normalized to LF in 20 week quad (n = 6).

Data are mean + SEM. *p < 0.05; **p < 0.01; ***p < 0.001 within groups and #p < 0.05; ##p < 0.01; ###p < 0.001 compared to LF. Different letters represent significant difference between groups in western blots. BCAA, branched-chain amino acids; Val, valine.
