# Supplementary figures and images for: Detrimental effects of branched-chain amino acids in glucose tolerance can be attributed to valine induced glucotoxicity in skeletal muscle
Source: Nutr Diabetes. 2022 Apr 13;12:20. doi: 10.1038/s41387-022-00200-8 (PMC9008040; doi:10.1038/s41387-022-00200-8)

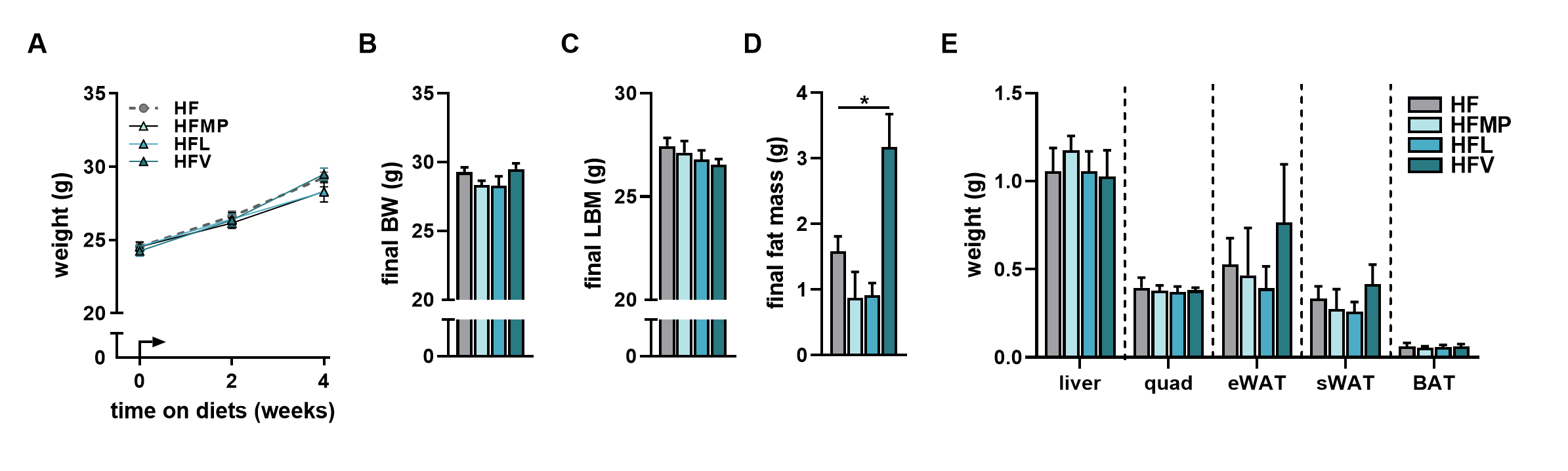

Supplement: Supplementary file 2 — Supplementary Figure 1 [file 41387_2022_200_MOESM2_ESM.tif]

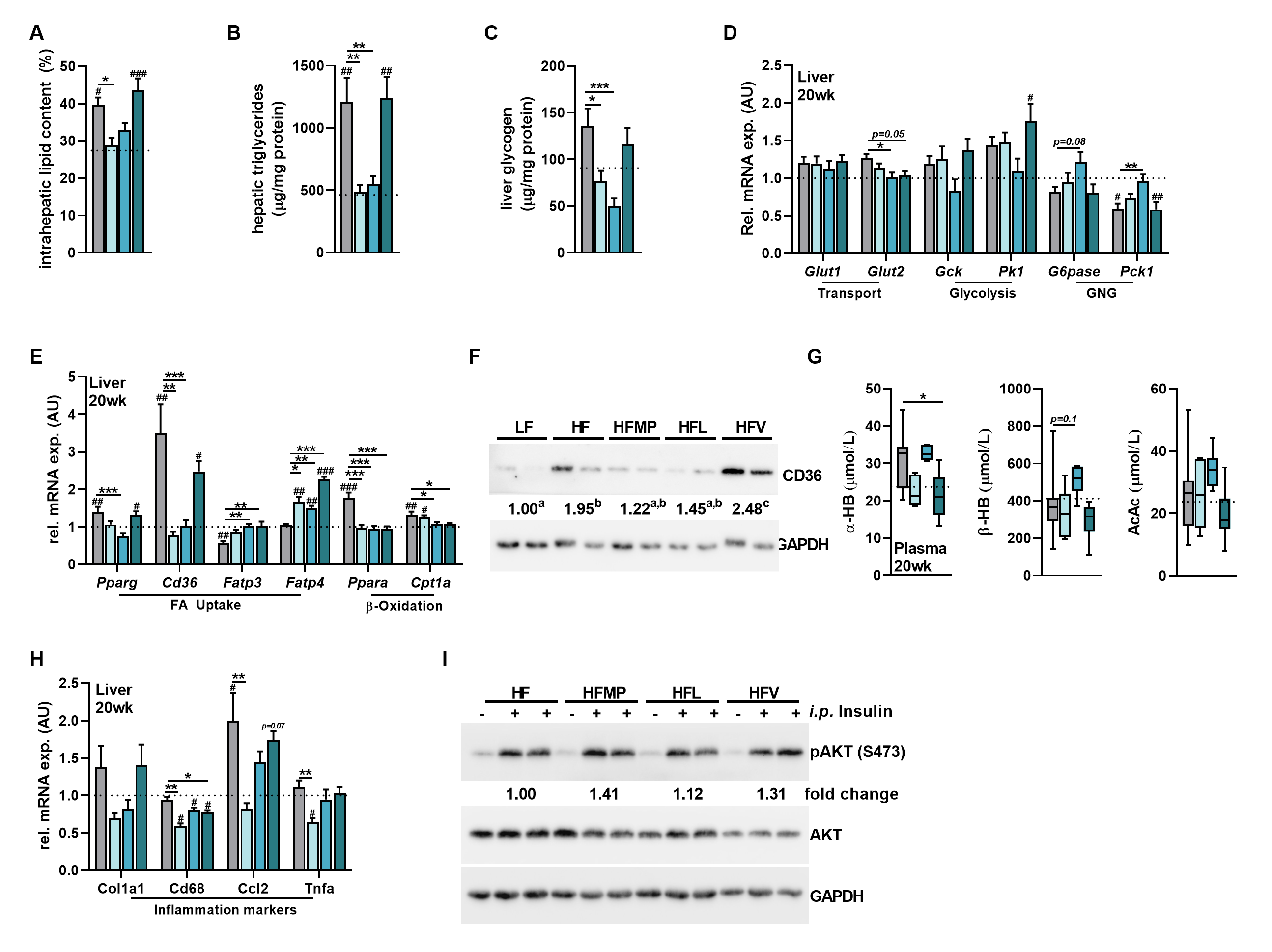

Supplement: Supplementary file 3 — Supplementary Figure 2 [file 41387_2022_200_MOESM3_ESM.tif]

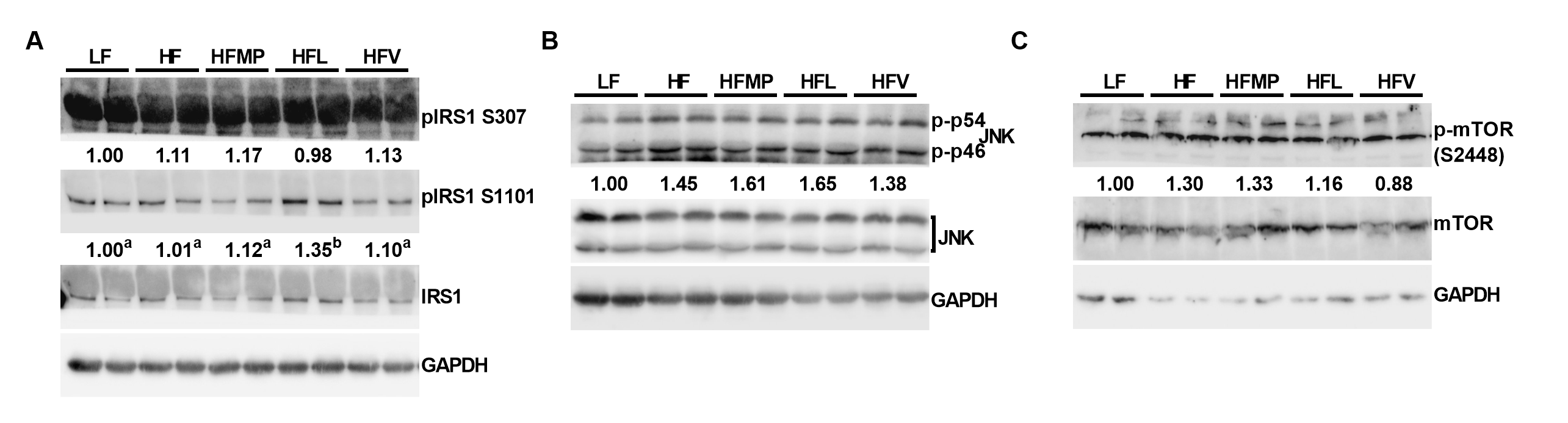

Supplement: Supplementary file 4 — Supplementary Figure 3 [file 41387_2022_200_MOESM4_ESM.tif]
